# Efficacy and safety of eribulin therapy for breast cancer with liver metastasis: a retrospective real-world study

**DOI:** 10.3389/fonc.2026.1795498

**Published:** 2026-05-12

**Authors:** Zishuo Wang, Xiaodong Xu, Pengwei Lv, Jianxiang Zhang

**Affiliations:** Department of Breast Surgery, The First Affiliated Hospital of Zhengzhou University, Zhengzhou, Henan, China

**Keywords:** breast cancer, eribulin, liver metastases, metastatic breast cancer, objective response rate, progression-free survival, real-world study, retrospective study

## Abstract

**Background:**

Eribulin is a microtubule inhibitor indicated for metastatic breast cancer (MBC). Previous studies have demonstrated its overall survival benefit in patients with MBC, but data on its efficacy in those with liver metastases are lacking.

**Methods:**

We retrospectively analyzed the efficacy and safety of eribulin in 73 patients with liver metastases from breast cancer treated at the First Affiliated Hospital of Zhengzhou University between 2020 and 2024. Systemic progression-free survival and objective response rate were assessed per RECIST version 1.1, and adverse events were evaluated.

**Results:**

The median progression-free survival was 3.7 months (95% CI: 2.8–4.6), with a systemic objective response rate of 15.1% (95% CI: 7.8%–25.4%; partial responses only). The highest objective response rate (21.2%) was numerically observed in the HR+/HER2− subtype. The most common adverse events were neutropenia (42.5%), fatigue (37.0%), alopecia (34.2%), and peripheral neuropathy (28.8%), with no treatment-related deaths.

**Conclusions:**

In this single-center retrospective exploratory analysis, eribulin-based therapy demonstrated preliminary activity in heavily pre-treated breast cancer patients with liver metastases. These hypothesis-generating findings warrant further prospective validation.

## 1 Introduction

Breast cancer is the most common malignancy among women worldwide (1). The incidence of breast cancer has steadily increased over recent decades in China, where it is now the most frequently diagnosed cancer in women and represents a major public health concern (2). The global burden is projected to rise to 3.2 million new cases and over 1.1 million deaths annually by 2050 (3). Notably, about 50%–60% of patients with metastatic breast cancer (MBC) develop liver metastases (4). Breast cancer liver metastases (BCLM) are typically associated with a high tumor burden, rapid disease progression, and a poor prognosis. Several studies have found that median overall survival (OS) is generally less than 2 years in patients with BCLM. For example, a population-based study found that patients with BCLM had a median OS of approximately 15 months, ranging from 8 months in triple-negative cases to 31 months in HR+HER2+ cases (5). Another large cohort analysis reported a median OS of 20.0 months using SEER (Surveillance, Epidemiology, and End Results) data and 27.3 months using FUSCC (Fudan University Shanghai Cancer Center) data (6). Notably, median OS was longer in patients who responded to chemotherapy or endocrine therapy than in those who did not (13 months vs 4.23 months) (7). Systemic therapy remains the mainstay of management for BCLM. A multicenter study found that HER2-targeted therapy significantly reduced the risk of mortality in patients with HER2+ disease, while surgical interventions such as liver resection provided additional survival benefits for selected patients but were typically combined with systemic treatment (8, 9).

Eribulin is a non-taxane inhibitor of microtubule dynamics that binds to the plus ends of microtubules (10, 11), inhibiting their polymerization without affecting depolymerization (12), which leads to irreversible blockade of mitosis at the G2–M phase and apoptotic cell death (13). In addition to its direct cytotoxic activity, eribulin has been shown to induce remodeling of the tumor vasculature and to improve tumor perfusion (14), and reverse epithelial–mesenchymal transition (15), thereby potentially enhancing drug delivery and inhibiting metastatic spread. Clinically, eribulin has demonstrated significant survival benefits in patients with MBC, who have been treated with anthracycline-based and taxane-based therapies previously (16). These unique mechanisms distinguish eribulin from other microtubule-targeting agents and support its use as an effective therapeutic option in advanced disease settings.

Previous research has found that eribulin extends OS in patients with MBC (17). Although eribulin increases the possibility of a favorable outcome, there is still a lack of targeted research on its therapeutic effect in patients with metastases to specific organs, such as the liver. The aim of this study was to determine the effectiveness and safety of eribulin in patients with BCLM.

## 2 Materials and methods

### 2.1 Patients

This retrospective study included women aged ≥18 years with a histopathologically confirmed diagnosis of primary breast cancer who were treated at the First Affiliated Hospital of Zhengzhou University between 2020 and 2024. The presence of liver metastases was confirmed by radiological imaging, including contrast-enhanced computed tomography (CT) and/or magnetic resonance imaging (MRI), in accordance with routine clinical practice. Patients were required to have received at least one cycle of eribulin-based chemotherapy during the study period to be included in the safety population. Patients with incomplete clinical records, those with no follow-up imaging for evaluation of tumor response, and those with unclear pathological or metastatic status were excluded from the efficacy analysis. Hormone receptor (HR) status, HER2 status, and Ki-67 proliferation index were determined by immunohistochemical analysis of tumor specimens. HER2 2+ (equivocal) cases were assessed further using fluorescence *in situ* hybridization. Patients with liver metastases originating from other primary malignancies, including ovarian cancer, colorectal cancer, gastric cancer, or other non-breast tumors, were excluded, as were those with synchronous or metachronous malignancies unrelated to breast cancer. Baseline demographic and clinical characteristics, including age, molecular subtype, previous systemic treatments, and metastatic burden, were extracted from the electronic medical records system. Written informed consent was obtained from all patients before inclusion in the study. A detailed breakdown of excluded patients is provided in **Supplementary Table 1**.

### 2.2 Ethics approval

This study was approved by the Research Ethics Committee of the First Affiliated Hospital of Zhengzhou University (Ethics Approval Number:2025-KY-1605-001).

### 2.3 Data retrieval

The relevant data for each patient were collected from our electronic medical records system. Tumor grade and stage were defined according to the Tumor–Node–Metastasis classification system devised by the International Union Against Cancer, and histology was classified according to the World Health Organization standard. Information on estrogen receptor, progesterone receptor, and HER2 status and the Ki-67 proliferation index was obtained from the pathology reports. The HER2 grading was as follows: 0 or 1+, negative, 2+, equivocal, and 3+, positive. Fluorescence *in situ* hybridization was performed for HER2 2+ samples to confirm HER2 amplification. Tumor response was assessed systemically according to Response Evaluation Criteria in Solid Tumors (RECIST) version 1.1. Target lesions in the liver were measured by abdominal MRI or CT, while extra-hepatic disease was assessed by chest CT, bone scan, or brain MRI as clinically indicated. The overall systemic response incorporated findings from all disease sites. Patients were required to have at least one measurable lesion per RECIST v1.1 at baseline to be eligible for efficacy analysis. Information on adverse events that occurred during treatment was also collected.

### 2.4 Eribulin treatment regimen

Eribulin was administered at a dose of 1.4 mg/m² via intravenous infusion on days 1 and 8 of each 21-day treatment cycle in accordance with the standard regimen. Dose modifications, including treatment delays or dose reductions to 1.1 mg/m² and subsequently to 0.7 mg/m², were permitted in cases with clinically significant adverse events, such as grade 3–4 hematologic or non-hematologic toxicity. Eribulin treatment was stopped once disease progression, unacceptable toxicity, or patient refusal to continue with treatment. For HER2-positive patients, eribulin was administered in combination with anti-HER2 monoclonal antibody therapy (trastuzumab) as continuation of HER2 blockade. A subset of HER2-negative patients received eribulin in combination with anti-angiogenic agents or immunotherapy at the discretion of the treating physician. The majority of patients received eribulin without concurrent cytotoxic chemotherapy. A small proportion of patients received eribulin in combination with another cytotoxic agent (capecitabine or carboplatin) at the discretion of the treating physician. Details of all combination regimens are provided in the footnote to **Table 1**.

**Table 1:** Patient demographic and clinical characteristics.

Characteristic	No. of patients (%)
Age(years)	
Median(range)	51(25-79)
Hormone receptor status*	
Positive	33(45.2)
HER2 status**	
Positive (IHC 3+ or 2+ and FISH+)	17(23.3)
Triple-negative (HR- and HER2-)	23(31.5)
Metastatic site	
Liver	73(100)
Bone	40(54.8)
Lung	17(23.3)
Brain	17(23.3)
Lymph node	16(21.9)
Eribulin regimen	
Monotherapy	40(54.8)
Combination***	33(45.2)
Line of Eribulin therapy	
≤3^rd^-line	51(69.9)
≥4^th^-line	22(30.1)

*Hormone receptor-positive was defined as estrogen receptor-positive and/or progesterone receptor-positive by IHC, with HER2 negativity. **HER2 positivity was defined as an IHC result of 3+ or as 2+ with positive FISH amplification, irrespective of hormone receptor status. FISH, fluorescence *in situ* hybridization; HR, hormone receptor; IHC, immunohistochemistry ***Among the 33 patients who received combination therapy, all HER2-positive patients received concomitant anti-HER2 targeted agents (trastuzumab, pertuzumab, or other anti-HER2 monoclonal antibodies, with or without HER2 tyrosine kinase inhibitors such as pyrotinib or neratinib). Among the HER2-negative patients, the majority received eribulin with anti-angiogenic agents (e.g., anlotinib) and/or immune checkpoint inhibitors (e.g., camrelizumab, sintilimab); a small number received eribulin in combination with another cytotoxic agent (capecitabine or carboplatin). Two patients received bone-modifying agents (denosumab or zoledronic acid) for bone metastases.

### 2.5 Statistical analysis

The primary outcomes of this study were systemic objective response rate (ORR) and progression-free survival (PFS). PFS was defined as the time from initiation of eribulin-based therapy to radiographic disease progression at any site (hepatic or extra-hepatic) per RECIST v1.1, or death from any cause, whichever occurred first. ORR was defined as the proportion of patients achieving a complete response (CR) or partial response (PR) in all measurable target lesions per RECIST v1.1. Patients without documented progression were censored at the date of their last adequate tumor assessment.

Given the retrospective, exploratory nature of this study with a limited sample size, the statistical analysis was primarily descriptive. Progression-free survival was estimated using the Kaplan–Meier method and presented with 95% confidence intervals. Objective response rates were reported as point estimates with corresponding 95% confidence intervals calculated using the Clopper–Pearson exact method. No formal statistical comparisons between subgroups were performed due to limited sample size and the descriptive nature of the analysis. All statistical analyses were performed using SPSS version 26.0 (IBM Corp., Armonk, NY, USA).

## 3 Results

### 3.1 Patient characteristics

**Figure 1:**
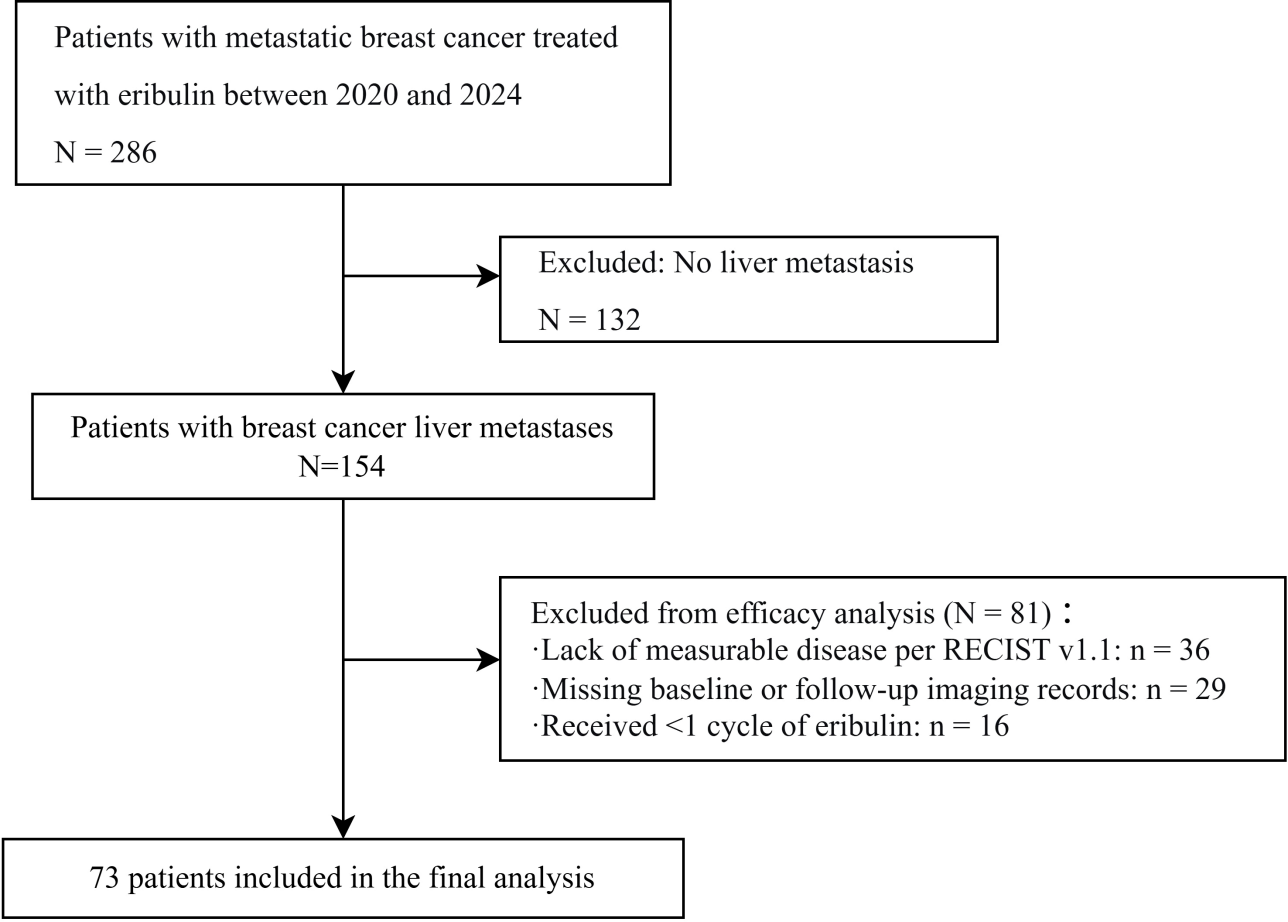
Flowchart showing the process used to select the study participants.

In total, 286 patients with metastatic breast cancer received eribulin in the Department of Breast Surgery at The First Affiliated Hospital of Zhengzhou University during the study period. Among these, 154 patients had confirmed liver metastases. After applying the predefined eligibility criteria, 73 patients with complete imaging records and measurable liver lesions were included in the final analysis (**Figure 1**). The patient demographic and clinical characteristics are summarized in **Table 1**. The median patient age was 51 years (range, 25–79). Various systemic therapies had been administered before eribulin. The pathological subtype of breast cancer was identified by immunohistochemistry. The distribution of pathology-based subtypes was as follows: HR-positive/HER2-negative (HR+/HER2−), n = 33 (45.2%); HER2-positive, n = 17 (23.3%); and triple-negative, n = 23 (31.5%). Twenty of the patients had isolated liver metastases and 53 had multiple metastatic sites involving the liver.

### 3.2 Efficacy of eribulin

**Figure 2:**
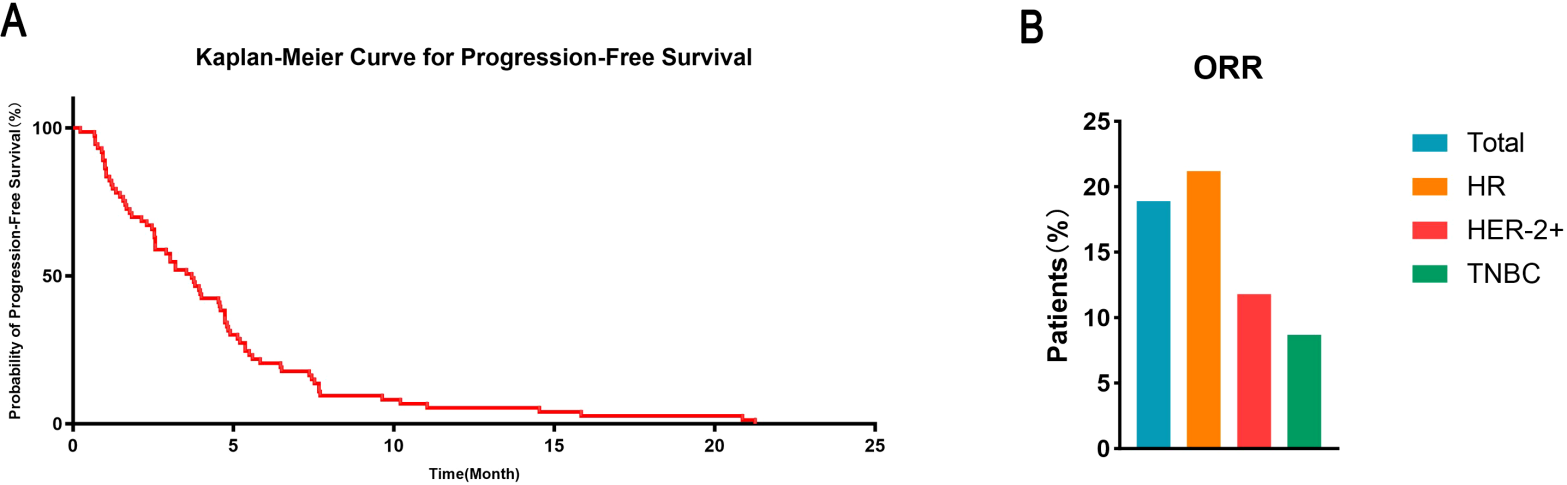
Efficacy of eribulin-based therapy. **(A)** Kaplan–Meier curve for progression-free survival in the overall cohort (N = 73). PFS was defined as time to systemic progression per RECIST v1.1 or death. **(B)** Systemic objective response rate per RECIST v1.1 in the total study population and by molecular subtype.

Median PFS was 3.7 months (95% CI: 2.8–4.6) after initiation of eribulin-based therapy. The systemic ORR per RECIST v1.1 was 15.1% (11/73; 95% CI: 7.8%–25.4%), with all responses being partial. Tumor responses by molecular subtype are shown in **Table 2**. Numerically, the HR+/HER2− cohort showed the highest ORR (21.2%; 7/33), followed by the HER2-positive cohort (11.8%; 2/17) and the triple-negative cohort (8.7%; 2/23). **Figure 2** shows the Kaplan–Meier curve for PFS and the ORR by subtype. An exploratory subgroup analysis of PFS by treatment regimen (monotherapy vs. combination therapy) is provided in **Supplementary Figure 1**.

**Table 2:** Tumor response to eribulin by molecular subtype in 73 patients.

Response	Total n = 73	HR(+)/HER2(-) n = 33	HER2(+) n = 17	Triple negative n = 23
CR	0	0	0	0
PR	11	7	2	2
Objective Responses (n)	11	7	2	2

CR, complete response; HR, hormone receptor; ORR, objective response rate.

### 3.3 Safety of eribulin

All 73 patients were included in the safety analysis. The most common treatment-emergent adverse events (any grade) were neutropenia (42.5%), fatigue (37.0%), alopecia (34.2%), and peripheral neuropathy (28.8%) (**Table 3**). Nausea occurred in 20.5% of patients and febrile neutropenia and infection in 4.1% each. Hepatic dysfunction was uncommon (1.4%). The most common grade 3–4 adverse events were neutropenia (31.5%) and fatigue (11.0%). Dose reductions because of toxicity were required in 17.8% of patients. Treatment was discontinued in 8.2% of patients because of adverse events, most of which were persistent hematologic toxicity or peripheral neuropathy. No treatment-related deaths occurred during the study period, indicating that eribulin had a manageable and clinically acceptable safety profile in this heavily pretreated population.

**Table 3:** Treatment-emergent adverse events in 73 patients treated with eribulin.

Adverse Event	Total grade n(%)	Grade 3–4 n(%)	Dose reduction n(%)	Treatment discontinuation n(%)
Neutropenia	31(42.5)	23(31.5)	6(8.2)	2(2.7)
Fatigue	27(37.0)	8(11.0)	5(6.8)	2(2.7)
Alopecia	25(34.2)	0(0.0)	0(0.0)	0(0.0)
Peripheral neuropathy	21(28.8)	5(6.8)	3(4.1)	1(1.4)
Nausea	15(20.5)	3(4.1)	2(2.7)	1(1.4)
Febrile neutropenia	3(4.1)	2(2.7)	0(0.0)	0(0.0)
Infection	3(4.1)	1(1.4)	0(0.0)	0(0.0)
Hepatic dysfunction	1(1.4)	1(1.4)	0(0.0)	0(0.0)
Total	52(71.2)	28(38.4)	13(17.8)	6(8.2)

### 3.4 Comparison across treatment lines

Eribulin-based therapy was administered as up to third-line therapy in 51 patients (69.9%) and as fourth-line or later in 22 patients (30.1%). Median PFS was 3.7 months (95% CI: 2.6–4.8) in patients receiving treatment as up to third-line and 3.2 months (95% CI: 1.9–4.5) in those treated in later lines. Given the limited sample size and the descriptive nature of this analysis, formal statistical comparison between these subgroups was not performed.

## 4 Discussion

This study retrospectively evaluated the efficacy and safety of eribulin in the routine treatment of patients with BCLM. Our findings demonstrated notable ORR and PFS. Among the molecular subtypes, patients with the HR+/HER2− subtype of breast cancer showed the highest ORR (21.2%), while the triple-negative subtype achieved an ORR of 8.7%. In this exploratory descriptive analysis, numerical differences in ORR were observed across molecular subtypes; however, formal statistical comparison was precluded by the limited sample size and the descriptive nature of the study. Activity was observed across different lines of therapy, although the independent impact of prior treatment exposure cannot be isolated from this dataset. The observed activity of eribulin in this heavily pre-treated cohort is broadly consistent with the survival benefit demonstrated in the pivotal EMBRACE trial, which established eribulin as a standard later-line option in metastatic breast cancer (17), and with subsequent real-world studies (18). Collectively, these data support eribulin as an effective therapeutic option for these patients.

In terms of safety, the adverse event profile in our cohort was generally consistent with the known toxicity spectrum of eribulin reported in previous clinical trials (19). The most frequent treatment-related adverse events were neutropenia (42.5%), fatigue (37.0%), alopecia (34.2%), and peripheral neuropathy (28.8%), most of which were mild to moderate in severity. Grade 3–4 adverse events occurred in 38.4% of patients, primarily neutropenia (31.5%) and fatigue (11.0%). Dose reductions (17.8%) were implemented in some patients to manage toxicity, while permanent discontinuation was required in only 8.2% of cases. There were no treatment-related deaths. These findings indicate that eribulin has a manageable safety profile even in heavily pretreated patients, and most adverse events can be effectively controlled by appropriate dose adjustment and supportive care. This tolerability pattern aligns with that reported in the pivotal EMBRACE trial (17) and Study 301 (16), which also demonstrated that eribulin is generally well tolerated, with hematologic toxicities being the most common and reversible upon dose modification.

This study has several important limitations. First, the retrospective, single-arm design precludes causal inference and may have introduced selection bias. Second, the high exclusion rate (81/154, 52.6%) due to stringent RECIST criteria requiring measurable disease and complete imaging records may further limit generalizability, although baseline characteristics were comparable between included and excluded patients (**Supplementary Table 2**). Third, the predominance of combination therapy (45.2%) in this cohort precludes isolation of the independent contribution of eribulin from that of concomitant targeted agents; an exploratory subgroup analysis is provided in **Supplementary Figure 1**. Fourth, the small sample size (N = 73) limits statistical power and precludes meaningful multivariable adjustment for potential confounders. Fifth, mature overall survival data are not yet available, limiting assessment of long-term benefit. Accordingly, these results should be interpreted as exploratory and hypothesis-generating only, intended to inform the design of future prospective studies rather than to guide clinical practice (20, 21).

Although specific data on liver metastases from breast cancer are scarce, our results align with the subgroup analyses in recent studies (22, 23) and suggest that eribulin remains an effective therapeutic option for patients with MBC and a heavy visceral metastatic burden, including those with liver involvement. The numerical variation in response rates observed among molecular subtypes in our cohort is consistent with previous reports (24). The potential activity of eribulin-based therapy in the HR+/HER2− subtype warrants prospective investigation.

In conclusion, this single-center retrospective exploratory analysis demonstrates that eribulin-based therapy has preliminary activity and a manageable safety profile in heavily pre-treated breast cancer patients with liver metastases. These hypothesis-generating findings warrant confirmation in a dedicated prospective controlled study.

## Data Availability

The raw data supporting the conclusions of this article will be made available by the authors, without undue reservation.
