# Supplementary material for: Efficacy and safety of eribulin therapy for breast cancer with liver metastasis: a retrospective real-world study
Source: Front Oncol. 2026 May 12;16:1795498. doi: 10.3389/fonc.2026.1795498 (PMC13201134; doi:10.3389/fonc.2026.1795498)
Supplement: Supplementary file 1 [file DataSheet1.pdf]

Supplementary files

Supplementary Table S1. Reasons for exclusion from the liver-metastatic cohort

Reason for Exclusion	N	%
Lack of measurable disease per RECIST v1.1	36	44.4%
Missing baseline or follow-up imaging	29	35.8%
Received ＜1 cycle of Eribulin	16	19.6%
Total Excluded	81	100%

Supplementary Table S2. Comparison of baseline characteristics between included and excluded patients.

Characteristic	Included (N=73)	Excluded (N=81)	P-value
Median age (years)	51	53	0.07
HER2+	17(23.3%)	19(23.5%)	0.98
HR+	33(45.2%)	35(43.2%)	0.80
Median prior lines	3.1	3.2	0.57

Supplementary Table S3 Concordance between hepatic and systemic best response per RECIST v1.1

Hepatic Best Response	Systemic Best Response	N
CR	CR	-
PR	PR	11/11(100%)
SD/PD	SD/PD	62/62(100%)
Total		73/73(100%)

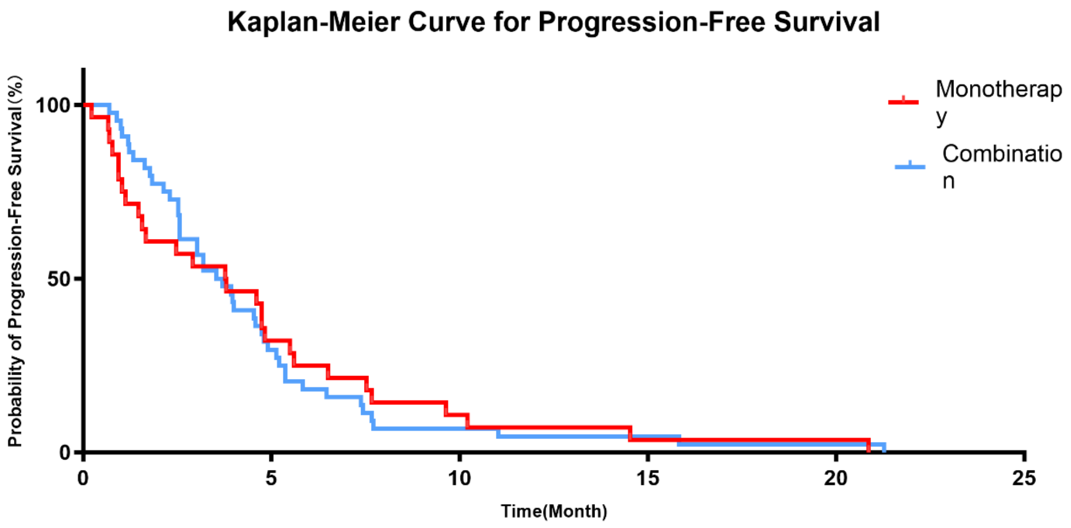

Supplementary Figure S1.Kaplan–Meier curves for progression-free survival stratified by treatment regimen (monotherapy vs. combination therapy). Kaplan–Meier estimates of PFS in patients receiving eribulin monotherapy (n=40) versus eribulin-based combination therapy (n=33). PFS was defined as time from initiation of eribulin-based therapy to systemic disease progression per RECIST v1.1 or death from any cause. Given the descriptive and exploratory nature of this analysis and the imbalanced subgroup sample sizes, formal statistical comparison between groups was not performed
